# Editorial: Computational intelligence advances in educational robotics

**DOI:** 10.3389/frobt.2023.1150409

**Published:** 2023-02-03

**Authors:** Francisco Bellas, Armando Sousa

**Affiliations:** ^1^ Integrated Group for Engineering Research, CITIC Research Center, Universidade da Coruña, Coruña, Spain; ^2^ Faculdade de Engenharia da Unviersidade do Porto, Porto, Portugal; ^3^ INESC TEC—INESC TEC Technology and Science, Porto, Portugal

**Keywords:** educational robotics, artificial intelligence—AI, computational thinking, programming skills, social robots

## 1 Introduction

The use of robots as educational tools at pre-university levels of education has become very popular (Daniela, 2019). Such tools are often also convenient in higher education, namely in the teaching of robotics in several areas of STEM. Robots are perfect for a learn-by-doing methodology, and they continue to be very motivating for younger students (Myint Swe Khine, 2017). Notably, younger students in higher education institutions also particularly value the visual learning style that such robots easily provide. The main reasons for the popularity of robots in schools (and higher education institutions) have been an increase in the availability of open software and open hardware options at affordable prices, and the development of programming environments suitable for students of different ages and skill levels (Esposito, 2017). Educational robots have been validated as effective ways to train students in programming fundamentals (Ioannou and Makridou, 2018), electronics and mechanics (Takacs et al., 2016), and more integrated STEM subjects (Tuluri, 2017).

Given the abovementioned context, robots can assist in education, specifically in the STEM areas, at various levels of teaching, starting from very young ages (pre-school) and extending all the way up to higher education.

We are currently entering the Artificial Intelligence education era (AIed) (Zimmerman, 2018); (Miao and Holmes, 2021). In this domain, students of all levels will require a formal training in AI Research Topic in the short term, and robots are perfect tools for this goal. They can be used as practical devices in class, where they can help students learn about perception, actuation, learning, reasoning, natural interaction, and many other Research Topic (AI4K12 initiative, 2021); (Developing an Artificial Intelligence, 2021). In addition, teachers can use robotic simulators in online learning scenarios, and for optimization of in-person class hours (Tselegkaridis and Sapounidis, 2021).

Regarding the term “computational intelligence,” the well-known Wikipedia site states that « there is still no commonly accepted definition of Computational Intelligence», but states that work in this field seeks the same long-term goal as AI (Wikipedia contributors, 2023): that is, to understand and learn to perform complex intellectual human tasks. The IEEE defines computational intelligence (CI) as «the theory, design, application and development of biologically and linguistically motivated computational paradigms. Traditionally the three main pillars of CI have been Neural Networks, Fuzzy Systems and Evolutionary Computation […] in addition to the three main constituents, it encompasses computing paradigms like ambient intelligence, artificial life, cultural learning, artificial endocrine networks, social reasoning, and artificial hormone networks. CI plays a major role in developing successful intelligent systems, including games and cognitive developmental systems. […] In fact, some of the most successful AI systems are based on CI» (IEEE Computational Intelligence Society, 2023).

Hence, in order to advance scientific knowledge in the broad area of AI, researchers and educators will be required to develop new resources focusing on AI and on CI, which will enable training on these Research Topic to be provided by robots. However, it is crucial that such studies and proposals go beyond particular tests in very specific domains with low transferability. As observed above, the usefulness of robots for education has already been validated; now, it is time to develop formal resources that can help the educational community to advance in digital education. In this way, not only educators but also policymakers will have reliable materials and studies enabling them to include AI education in their programs.

The particulars of the implementation of education involving robotics are also important to consider, because the increasing use of robots in teaching (whether telepresence robots or other types) will provide a larger number of students with a greater awareness of AI and robotic technologies. When students have hands-on experience of AI and robots, they are likely to be more knowledgeable and more aware of the pros and cons of such high-tech pieces of software and hardware, which will soon become natural components of our daily routines.

## 2 Included articles

The articles included in this Research Topic focus on two main Research Topic within AI education: computational thinking and social robotics.

Janika Leoste and her colleagues from Tallin University of Technology present an article about telepresence robots. They perform an exploratory piece of research with the aim of obtaining evidence on the perceptions of personnel from eight Georgian universities as to the role of telepresence robots in enhancing learning and teaching, and their views on the characterization of these robots in terms of challenges, benefits, opportunities, weaknesses, and threats.

Enrico Prosperi and colleagues from the Sapienza University in Rome present an article focusing on therapeutic education (TE) with robots. The aim of the research study described in their article is to evaluate the effect of employing a social robot as a therapeutic educational robot to help the expert therapist in an educational activity.

Regarding computational thinking, Francesca Bertacchini and her colleagues from the University of Calabria present a paper with the goal of making the functions of the NAO robot as social and autonomous as possible, adopting the Wolfram Language (WL) used in Mathematica software in their design process. In a similar vein, Matthias Funk and colleagues from University of the Azores present a review in which they analyze solutions proposed for the use of tangible programming languages (TPLs) in different contexts, combining these with non-TPL solutions such as graphical programming languages (GPLs). Various cognitive aspects of WL programming are also investigated.

## 3 Conclusion

The variety of considerations underlying AI and CI is enormous and will have relevant implications for today’s society. Care must be taken regarding education in such Research Topic for all ages, a particularly challenging Research Topic in a world where education is sometimes seen as lagging behind other sciences. The current volume contributes new information to this area.
